# Transactions of the Medical Society of Pennsylvania

**Published:** 1878-04

**Authors:** 


*Transactions of the Medical Society of the State of Pennsylvania,* at its Twenty-eighth Annual Session, held at Harrisburgh, June 13th, 1877.

The plan of this volume is different somewhat from that of a similar one issued by the Medical Society of the State of New York whose sessions are mainly occupied with the President's address and the reading and discussions of original papers of considerable number and merit, which are voluntarily communicated by delegates. The Pennsylvania State Society at its annual session has an address by the President and also addresses in the departments of Medicine, Obstetrics, Surgery, Hygiene and Mental Disorders by regular appointees and the presentation and discussion of original communications of which the report for the last meeting contains but six. Following report of these, the volume presents selected reports from eighteen county societies of matters of general reference to a locality or of note-worthy cases or experience reported by members of County Societies and recommended by the

County Society to the Standing Committee of the State Society for examination, discussion or publication as the letter may direct. In this way two hundred pages of material are obtained for the volume which is thus made of more particular interest to physicians of different localities and some desirable material brought forth. The plan is an excellent one for County Societies as it encourages more general report of individual experience, and greater ability in observation is likely to be developed by it.

The addresses contained in the volume are quite meritorious. S. B. Kiefer, A. M., M. D., treats under this head, of Puerperal Convulsions; John Curwen, M. D., of Mental Disorders quite interestingly and of length; Benj. Lee, A. M., M. D., of Hygiene; and H. L. Hodge, M. D., of Surgery, giving a very excellent résumé of the latest advances in this department. An original communication by P. D. Keyser, M. D., of Wills Ophtholinic Hospital presents cases of conjunctivitis, blepharitis and pterygium which he considers arose from defects of refraction and accomodation. Robert B. Mowey, M. D., in his Presidential address makes the following statement in reference to an address lately delivered before the Royal Microscopical Society :

"Mr. Loeby belives that the visibility of minute objects with the best microscopes now constructed is limited to an object, the size of which would be between the 1, 80.000th and the 1, 100.000th of an inch; he then says that, according to the mathematics of Sir Wm. Thomson, there is contained in the 1, 80.000th of an inch about 2.000 molecules of water, and therefore in order to see these molecules, it would be necessary to use a magnifying power of from 500 to 2.000 times greater power than the best now in use; and if we could construct one of such high powers, it could not be used, unless the waves of light were some 1, 2.000th part the length they are now supposed to be, and our eyes were also correspondingly improved in perfection."

